# Major iatrogenic bile duct injury during elective cholecystectomy: a Czech population register-based study

**DOI:** 10.1007/s00423-023-02897-2

**Published:** 2023-04-20

**Authors:** Dušan Klos, Michal Gregořík, Tomáš Pavlík, Martin Loveček, Jana Tesaříková, Pavel Skalický

**Affiliations:** 1 Department of Surgery I., Faculty of Medicine and Dentistry, University Hospital Olomouc and Palacký University Olomouc, Zdravotníků 248/7, CZ-77900 Olomouc, Czech Republic; 2 Institute of Health Information and Statistics of the Czech Republic, Palackého náměstí 4, CZ-12801 Prague, Czech Republic; 3 Faculty of Medicine, Institute of Biostatistics and Analyses, Masaryk University, Kamenice 753/5, CZ-62500 Brno, Czech Republic

**Keywords:** Bile duct injury, Cholecystectomy, Critical view of safety, Postoperative complications

## Abstract

**Purpose:**

Bile duct injury (BDI) remains the most serious complication following cholecystectomy. However, the actual incidence of BDI in the Czech Republic remains unknown. Hence, we aimed to identify the incidence of major BDI requiring operative reconstruction after elective cholecystectomy in our region despite the prevailing modern 4 K Ultra HD laparoscopy and Critical View of Safety (CVS) standards implemented in daily surgical practice among the Czech population.

**Methods:**

In the absence of a specific registry for BDI, we analysed data from The Czech National Patient Register of Reimbursed Healthcare Services, where all procedures are mandatorily recorded. We investigated 76,345 patients who were enrolled for at least a year and underwent elective cholecystectomy during the period from 2018–2021. In this cohort, we examined the incidence of major BDI following the reconstruction of the biliary tract and other complications.

**Results:**

A total of 76,345 elective cholecystectomies were performed during the study period, and 186 major BDIs were registered (0.24%). Most elective cholecystectomies were performed laparoscopically (84.7%), with the remaining open (15.3%). The incidence of BDI was higher in the open surgery group (150 BDI/11700 cases/1.28%) than in laparoscopic cholecystectomy (36 BDI/64645 cases/0.06%). Furthermore, the total hospital stays with BDI after reconstruction was 13.6 days. However, the majority of laparoscopic elective cholecystectomies (57,914, 89.6%) were safe and standard procedures with no complications.

**Conclusion:**

Our study corroborates the findings of previous nationwide studies. Therefore, though laparoscopic cholecystectomy is reliable, the risks of BDI cannot be eliminated.

## Introduction

Elective cholecystectomy is the gold standard treatment for symptomatic cholelithiasis and its complications. Though a majority of cholecystectomies are performed laparoscopically, a smaller proportion is performed openly. Hence, a new robot-assisted cholecystectomy program is currently being developed [1, 2]. Laparoscopic cholecystectomy is a popular and routinely performed procedure in general abdominal surgery worldwide [3]. However, bile duct injury (BDI) is the most serious complication of laparoscopic cholecystectomy with an incidence of 0.2–0.7%, and significantly impacts increased morbidity, quality of life, and overall survival [4–6]. Additionally, BDI has significant medical-legal liabilities and frequently causes civil litigation involving personal injury claims.

Several systems are available for the classification of BDI. Bismuth´s classification [7] includes five types of bile lesions according to the distance from the hepatic hilus and involvement of the bile duct bifurcation. Type I involves the common bile duct and low common hepatic duct (CHD) > 2 cm from the hepatic duct confluence. Type II involves the proximal CHD < 2 cm from the confluence. Type III is a hilar injury with no intact residual CHD confluence. Type IV is the destruction of the confluence when the right and left hepatic ducts separate. Type V involves the aberrant right sectoral hepatic duct alone or with concomitant injury of the CHD. Strasberg proposed modification of this classification, including particular biliary leaks [8]. This modification allows differentiation between small (bile leakage from the cystic duct or aberrant right sectoral branch) and serious injuries caused during laparoscopic cholecystectomy as type A to E. Type E of the Strasberg classification with its subtypes (E1-E4) is analogous to the Bismuth classification. McMahon et al. [9] defined major bile duct injury as at least one of the following present: laceration of > 25% of bile duct diameter, transection of the CHD or common bile duct (CBD), or the development of a postoperative bile duct stricture. A minor bile duct injury, according to this classification is a laceration of the CBD of < 25% of the diameter or a laceration of the cystic-CBD junction ("buttonhole tear"). The Amsterdam Academic Medical Center´s classification by Bergman et al. [10] includes BDIs type A-D. Type A involves cystic duct leaks or leakage from aberrant or peripheral hepatic ductules; type B involves major bile duct leaks with or without concomitant biliary strictures; type C involves bile duct strictures without bile leakage, and type D is defined as a complete transection of the duct with or without excision of some portion of the biliary tree. Neuhaus et al. [11] described 5 types of BDIs (A-E): type A is a peripheral bile leak (in communication with the CBD), type B is an occlusion of the CBD (or right or left hepatic duct, ie clip, ligation), type C is a lateral injury of the CBD, type D is a transection of the CBD (or right hepatic duct not in communication with the CBD), and type E is stenosis of the CBD. Csendes et al. [12] classified BDIs as type I-IV. Type I is characterised as a small tear of the hepatic duct or right hepatic branch caused by dissection with the hook or scissors during the dissection of Calot´s triangle. Type II are lesions of the cysticocholedochal junction due to excessive traction, the use of a Dormia catheter, transection of the cystic duct very close to, or at the junction with the CBD, or burning of the cysticocholedochal junction by electrocautery. Type III is a partial or complete transection of the CBD, and type IV is a resection of more than 10 mm of the CBD. The following classification systems are a combination of BDI and vascular injury descriptions. The Stewart-Way classification [13] included four classes based on the combination of mechanism and localisation of BDI. Both complex bile duct and vascular injuries were including in this classification. Class I injury occurs when the CBD is mistaken for the cystic duct, but the error is recognised before the CBD is divided. Class II injuries involve damage to the CHD from clips or cautery used too close to the duct. This often occurs in cases where visibility is limited due to inflammation or bleeding. Class III injury, the most common type, occurs when the CBD is mistaken for the cystic duct. The common duct is transected, and a variable portion, including the junction of the cystic and common duct, is excised or removed. Class IV injuries involve damage to the right hepatic duct, either because this structure is mistaken for the cystic duct or because it is injured during dissection. The Hannover classification proposed by Bektas et al. [14] divided BDIs into five types from A to E. Type A is peripheral bile leakage, and type B is a stricture of the CHD or CBD without injury. Type C is lateral CHD or CBD injury. Type D is the total transection of the CHD. Type E is a stricture of the main bile duct without bile leakage postoperatively. Vascular injuries are included in types C and D. Lau’s classification [15] defined 5 types of injuries, and the most recent ATOM classification was published by the EAES in 2013 [16] using semantic connotations, grouped in three easy-to-remember categories, A (for anatomy), To (for time of), M (for mechanism): (1) the anatomic characteristics of the injury: NMBD for non-main bile duct or MBD for main bile duct (followed by a number 1–6, corresponding to the anatomic level on the MBD), followed by Oc (for occlusion) or D (division), P (partial) or C (complete), LS (loss of substance), VBI (vasculobiliary injury in general), and whenever known the vessel; (2) time of detection: Ei (early intraoperative), Ep (early postoperative) or L (late); and (3) mechanism of injury: Me (mechanical) or ED (energy-driven).

The best therapeutic outcome is intraoperative recognition of a BDI, followed by immediate reconstruction [17]. However, only ˂ 25–30% of BDIs are recognised during the primary operation] [18]. Late diagnosis is associated with the following clinical conditions: persistent or abnormal abdominal pain, bile leakage from the drainage, clinical signs of biliary peritonitis such as fever, jaundice, and elevated laboratory parameters of liver dysfunction and sepsis. Currently, CT abdominal scan is the first-choice method for diagnosing any intraperitoneal fluid collections and identifying associated vascular lesions primarily on the right hepatic artery. Magnetic resonance cholangiopancreatography (MRCP) is the benchmark for the morphological assessment of the biliary tree and its integrity [19]. Endoscopic retrograde cholangiopancreatography (ERCP) is a combined diagnostic and therapeutic treatment tool that is preferred as a conservative therapeutic approach for cystic duct leaks and Stewart-Way Class I and II injuries [20]. In cases of partial or complete transection of the common bile duct, a direct end-to-end suture of the distal choledochus without tension is recommended. Roux-en-Y hepaticojejunostomy is the first choice for reconstruction when direct suturing is not possible [21, 22].

The Critical View of Safety (CVS), first described in 1995, is considered to be the standard for the identification of cystic structures [23]. Three required criteria to achieve CVS are: the hepatocystic triangle must be cleared, the lower third of the gallbladder must be separated from the liver bed, and only two structures should be entering the gallbladder. Complementary imaging of the extrahepatic biliary tract anatomy includes intraoperative cholangiography (IOC) and indocyanine green fluoroscopy (ICGF). Complementary imaging may have a role in difficult cases; however, IOC is burdened by morbidity and mortality, and both IOC and ICGF possess limitations [24, 25]. Other options include consulting another surgeon and performing bail-out procedures such as open conversion, subtotal cholecystectomy, fundus-first, and tube cholecystectomy. These are included in the guidelines if CVS cannot be achieved [26, 27]. A general recommendation to prevent BDI is conversion to immediate open surgery in cases of complicated laparoscopic cholecystectomy. Standardisation of the CVS and its implementation in everyday surgical practice results in a reduction of the BDI rate from 0.32 to 0.23%, and even 0.09% if the CVS is properly implemented [23, 28, 29].

This novel study determines the current incidence of major BDIs during elective cholecystectomy (laparoscopic vs. open) in the era of modern 4 K Ultra HD laparoscopy and CVS implementation into daily surgical practice, including IOC and ICGF, in the Czech population.

## Material and methods

**Fig. 1:**
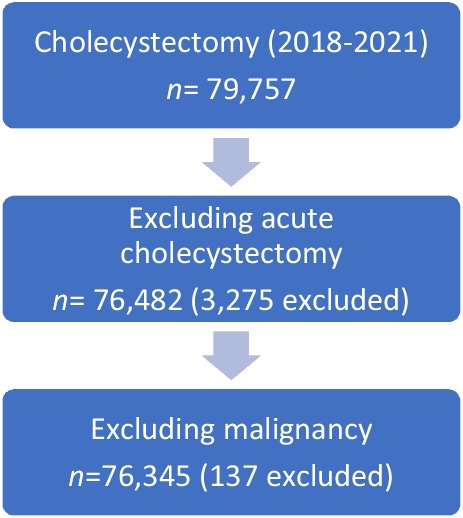
Flowchart of patient enrolment

All patients registered in the Czech National Patient Register of Reimbursed Healthcare Services 2018–2021 with a DRG system code indicating laparoscopic (90818) or open elective cholecystectomy (51371) were included in the study. In this cohort, a subset of patients was identified who had an open surgical bile duct reconstruction procedure after BDIs (DRG 51381 or 51377) reported within a single admission or readmission following a previous elective cholecystectomy for a single unique patient identifier number. (Fig 1)

The observation period was from 2018 to 2021, with an additional year of postoperative complication monitoring. Patients with acute cholecystectomies and cholecystectomies for malignancies were excluded from this study. The type of surgery (laparoscopic vs. open), the total number of procedures, the number of biliary tract reconstructions, the length of stay, and any type of complication within the DRG system (CC1-4) were obtained from the registries. Based on outcomes, all data were divided into two categories: elective laparoscopic vs. open cholecystectomy. This study was reported according to the STROBE statement.

### National register data

The Czech National Patient Register of Reimbursed Healthcare Services is a nationwide retrospective database that records every medical procedure within the public healthcare system in the Czech Republic, administered by the Czech Ministry of Health and our public healthcare insurance companies. Data are entered into this system by healthcare providers such as hospitals. Based on this data, reimbursement is obtained for the medical treatment provided. Laparoscopic or open elective cholecystectomy is performed under public health insurance and not on a private basis owing to legal restrictions under Czech law. Therefore, each surgical procedure and its postoperative complications must be recorded in this register.

### Definition of terms

#### Elective cholecystectomy

Elective cholecystectomy was defined as surgery for cholelithiasis and its complications after exclusion of acute cholecystectomy.

#### Acute cholecystectomy

Acute cholecystectomy was defined as an emergency procedure for cholelithiasis and its complications, as defined by the DRG marker for acute procedure and ICD-10 code K80.0, K80.3, K81.0, K82.2. Acute cholecystectomy was excluded from enrolling in this study cohort.

#### Major biliary injury

We chose to classify major BDIs as injury requiring operative repair, using perioperative DRG codes for hepaticojejunostomy or other biliary surgery ((DRG 51381 or 51377) as surrogates for BDI.

#### Postoperative complication

Other complications were identified using statistical coding within the Czech DRG system, classified as no complication CC0 or any type of complication CC1-4.

#### Length of in-hospital stay

LOS was defined as the total number of days in any hospital for complication related to the elective cholecystectomy.

### Statistical analyses

The collected data were statistically analysed using R software version 4.1.1. Study group (n)and % were analysed as qualitative variables, whereas for qualitative variables, the mean with standard deviation or median with the 1st and 3rd quartiles was employed. The normality of the distribution was evaluated using the Shapiro–Wilk test while the relationships between two categorical variables were assessed using Fisher’s exact test. To compare variations in continuous variables between the two groups, the Mann–Whitney U test was utilised.

### Ethics

The study was conducted in accordance with the Declaration of Helsinki and approved by the Ethics Committee of the University Hospital Olomouc (EKFNOL-19/2023). Owing to the retrospective nature of the study and the use of anonymous clinical data for analysis, informed consent was not required.

## Results

A total of 76,345 elective cholecystectomies were performed during the study period, and 186 major BDIs were registered (0.24%). The basic demographics are listed in Table 1. Most elective cholecystectomies were performed laparoscopically (84.7%), with the remaining conducted as open surgeries (15.3%). The mean age of the patients was 59.5 years, and the majority were women (66.2%).

**Table 1:** Patient demographics

Factor	Total	Laparoscopic	Open
*n* (%)	76,345 (100%)	64,645 (84.,7%)	11,700 (15.3%)
Mean age (STD)	60.3 ± 5.14	55.1 ± 6.2	65.4 ± 5.1
Male (%)	46.7	35.8	57.7
Female (%)	53.3	64.2	42.3

STD is standard deviation

### Bile duct injury

A total of 186 patients with major BDIs necessitating reoperation and bile duct reconstruction were identified. The incidence of BDI was higher in the open surgery group (150 BDI/11700 cases/1.28%) than in laparoscopic cholecystectomy (36 BDI/64645 cases/0.06%) (Tables 2 and 3). A minority of BDIs (15BDI/36 cases/41.7%) were detected during laparoscopic cholecystectomy. The majority of BDIs following laparoscopic cholecystectomy (21 BDI/36 cases/58.3%) were observed after re-admission. In open surgery, the maximum BDIs were detected during the operation (109 BDIs/ 150 cases/ 72.7%), and the remainder were detected after re-admission (41 BDIs/ 150 cases/ 27.3%). The total incidence of BDI after elective cholecystectomy was 0.24% (186 BDI/76,345 cases).

**Table 2:** Biliary tract injury characteristic

	Total *n* = 76,345	Laparoscopic *n* = 64,645	Open *n* = 11,700
BDI	186 (0.24%)	36 (0.06%)	150 (1.28%)
Admission	124 (66.6%)	15 (41.7%)	109 (72.7%)
Re-admission	62 (33.4%)	21 (58.3%)	41 (27.3%)

**Table 3:** Lenght of hospital stay and postoperative complications

	Laparoscopic	Open
LOS without BDI (days)	5.27	12.7
LOS with BDI (days)	N/A	13.6
No complication (*n*/%)	57,914 (89.6%)	9,664 (82.6%)
With complication (*n*/%)	6,731 (10.4%)	2,036 (17.4%)

### Length of in-hospital stay and other complications

The total hospital stay for patients with BDI after reconstruction was 13.6 days. The group with BDI during laparoscopic cholecystectomy had 12.7 days of hospital stay. Patients who underwent laparoscopic cholecystectomy without BDI were discharged from the hospital after 5.27 days, compared with 7.8 days after open surgery. The majority of laparoscopic elective cholecystectomies (57,914, 89.6%) were safe and standard procedures with no complications. Open surgery had more complications, and only 82.6% (9,664) of open cholecystectomies were completed without complications

## Discussion

To our knowledge, this is the first study to use data from healthcare insurance companies and hospitals that mandatorily perform cholecystectomies under the public healthcare system to estimate the number of BDIs in our region in central Europe. Cholecystectomy is a basic, safe, routine, and popular procedure in general surgery. However, bile duct injury, a serious complication of cholecystectomy may lead to late complications such as stenosis of the hepaticojejunostomy, followed by chronic cholangitis, biliary cirrhosis, portal hypertension, and liver failure. Studies indicate that the most common complications following bile duct reconstruction are recurrent cholangitis (11,4–23%), anastomotic leakage (5–10%), intra-abdominal abscess (14%), and anastomotic stricture (30%) [30–32]. Data from the Ministry of Justice of the Czech Republic, establish BDIs as the most common cause of legal liability, together with iatrogenic ureteral and recurrent laryngeal nerve injuries. The conditions for a court decision on financial compensation for the victim with BDI during an elective cholecystectomy depend on the: 1. surgery outside the established rules (cause), 2. adverse effects (BDI), and 3. causal link between cause and adverse effects. BDIs are the most common malpractice claims in gastrointestinal surgery and constitute half of the general surgery laparoscopic claims and 20% of all general surgery litigations in the US [33].

This study reported a decreased number of BDIs during elective laparoscopic cholecystectomies from the register. The incidence of the estimated BDI rate after elective cholecystectomy is 0.24%, however, after laparoscopic cholecystectomy, the BDI rate is reduced to 0.06%. In contrast, the study showed a relatively high incidence of BDIs in the open cholecystectomy group reported to this registry (1.29%). The low incidence of BDI in laparoscopic cholecystectomy in this study is something we are very critical of. This bias is attributed to the failure of recording the number of conversions from laparoscopic to open surgery in the national registry. Therefore, the open cholecystectomy section included a proportion of procedures that started as laparoscopic and continued as open but were only recorded as open cholecystectomy. Simple administrative error is another reason for the low incidence of BDI in the national registry. Healthcare providers deliberately underreport BDI during laparoscopic surgery, listing it as part of open cholecystectomy. The overall adjusted estimated incidence of BDIs following laparoscopic cholecystectomy in the Czech Republic is between 0.06–0.6%, based on our experience from a tertiary HPB centre in a region with a population of one million inhabitants.

This study also exhibited a relatively high rate of complications after the basic general surgical procedure. Complications were reported in 10.4% of patients after laparoscopic cholecystectomy and 17.4% after open cholecystectomy. The length of the hospital stay after uncomplicated cholecystectomy averaged 5.27 days after laparoscopic and 7.8 days after open cholecystectomy. The reason for the reported length of hospital stay is due to the imperfection of the diagnosis-related group (DRG) system in the Czech Republic and local practices (patients prefer longer hospital stays to shorter ones: one-day surgery). To be reimbursed for laparoscopic cholecystectomy at all, the minimum non-negotiable hospital stay is 3 days: first day, preparation; second day, surgery; third one, postoperative day 1. Since most local surgeons prefer using a drain that is removed 24 h after surgery, we have had already reduced hospital stay to 4 days. Considering that hospitals have little incentive to discharge patients early to maintain high levels of bed occupancy: such a length of time (5.27 days) might not be surprising. For bile duct repair after BDI, the average length of hospital stay was 12.7 days, which reflects the complexity of the procedure and its postoperative course. Complication rates for all cholecystectomies are as high as 8–12% and are significantly higher in cases of acute cholecystectomy [34].

The number of open procedures is relatively high, at 15.3%. Our hypothesis is that the main reason for the high number of open cholecystectomies is a simple clerical error, namely the lack of reporting of laparoscopy for initial and actual conversion. In our experience, the rate of laparoscopic cholecystectomy is reduced by the fact that many acute cholecystitis cases are treated conservatively, and cholecystectomy is delayed (particularly during the COVID-19 pandemic, when elective surgery was on lockdown). This is partly due to our senior colleagues preferring the conventional approach in selected patients (previous surgery, comorbidities, etc.).

We compared the data with other national studies as data on the incidence of BDI in the Czech Republic or a specific national registry does not exist. Small cohorts of patients with BDI and biliary reconstruction treated in specialised Czech HPB centres are available [32, 33, 35]. For example, Treska V. et al. [35] reported a cohort of 11 patients after biliary reconstruction for BDI with post-operative morbidity of 36.4% and mortality of 18.2%. However, there is no evidence of the incidence of BDI in our region. Hobbs et al. [36] stated that approximately 20 percent of all complications and 30 percent of BDIs were attributable to surgeons who had performed 200 or fewer cholecystectomies in the previous 5 years. Rystedt et al. [27] indicated that BDIs are associated with 55,134 cholecystectomies in Sweden with an incidence of BDI of 0.3%. The majority of BDIs in this national cohort were detected during cholecystectomy and were repaired by the operating surgeon. In the large US cohort specified by Barret et al. [26] the rate of BDI requiring surgery plateaued and remained at 0.23% despite increasing experience with laparoscopy. Moreover, cholecystectomy was associated with a 30-day morbidity rate of 9.84%. Therefore, in comparison with other studies, we conclude that the data from our national study are comparable with the results and incidence of BDI from cohorts of other large national studies.

The average BDI incidence rate after elective cholecystectomy was relatively low (0.24%). However, compared with other national studies, this rate has stagnated in recent years. This incidence rate not only has serious consequences for the quality of life of the patient but also multiplies the costs of complication management under the public health system. The Critical View of Safety has been recognised as the standard method for the identification of cystic structures to prevent vasculobiliary injuries during laparoscopic cholecystectomy [23]. National surveys have displayed very high rates of CVS application in certain countries (98% in the Netherlands, 97% in Japan, 84% in Korea, 82% in the UK, and Ireland) [37–39]. In the Czech Republic, CVS procedures are well adopted. Furthermore, the recommended SAGES Safe Cholecystectomy Programme procedure is used [32].

Causes of laparoscopic BDI are generally classified into technical errors during dissection and anatomical errors by the surgeon. Anatomical errors are more frequent (up to 85%) and usually involve misinterpretation of the common biliary duct or an aberrant right posterior sectional branch as the cystic duct [40, 41]. Obtaining a proper CVS requires an extensive clearance of the hepatocystic triangle, compared with the popular infundibular technique in cases of mild or no inflammation. Complementary imaging, including intraoperative cholangiography and indocyanine green fluoroscopy, are alternate options in difficult cases. When CVS cannot be achieved, the 2018 Tokyo, 2020 WSES, and RICARD guidelines recommend the exploration of alternate choices [23–25]. These include intraoperative complementary imaging, consultation with another surgeon, and bail-out procedures (open conversion, subtotal cholecystectomy, fundus-first cholecystectomy, and tube cholecystectomy). The learning curve for laparoscopic cholecystectomy has not yet been determined. However, a recent systemic review showed a significant discrepancy where included papers recommended between 13 and 200 cases for practicing laparoscopic cholecystectomy [42]. Training modules with a structured curriculum promote safety in laparoscopic cholecystectomy and increase the rates of successful CVS and improve confidence among trainees [43–45].

This study has many strengths, including mandatory data on a large nationwide scale. The database analysis has clear limitations. As this register is a medical-care-provider-driven system, only BDIs that required operative interventions were detected. However, BDIs that underwent endoscopic intervention, percutaneous transhepatic interventions, or resulted in patient death could not be assessed. Additionally, there are limitations to the definition of BDI between laparoscopic and open cholecystectomy. For some patients with BDI, laparoscopy is typically initiated, completed, and transferred to a tertiary HPB centre for definitive reconstruction. However, the procedure is already reported as open cholecystectomy with reconstruction. This explains the relatively low incidence of BDI in laparoscopic cholecystectomy and the high rate of open reconstruction on re-admission in our study. An administrative analysis of the database was performed in this study. Therefore, data on follow-up and complications are limited. However, to obtain real data on the incidence of BDIs during elective cholecystectomy and if no other type of national registry is available, the limitations of this study are acceptable.

## Conclusion

Iatrogenic BDIs are among the most serious complications of cholecystectomy. In this nationwide study, we demonstrated a low incidence of BDI during elective cholecystectomy according to the limitations of this study. Our study, which utilised data from the National Registry of Healthcare Providers, corroborates with the findings of previous nationwide studies. Laparoscopic cholecystectomy is a safe procedure owing to the adaptation of CVS principles and safety protocols. However, the risks of BDI cannot be eliminated. Therefore, any error by the surgeon and its causes must be carefully investigated. In the training of young surgeons, it is necessary to emphasise not only the importance of safety protocols during laparoscopic cholecystectomy but also the possibilities for the prevention, early diagnosis, and treatment of BDI. Thus, this analysis and the principles of CVS may lead to improvements in the quality of surgical care and a reduction in serious iatrogenic injury risks in patients.

## Data Availability

Data included in this study are available from the Ministry of Health.
